# Liver Retransplantation in Adults: The Largest Multicenter Italian Study

**DOI:** 10.1371/journal.pone.0046643

**Published:** 2012-10-05

**Authors:** Umberto Maggi, Enzo Andorno, Giorgio Rossi, Luciano De Carlis, Umberto Cillo, Fabrizio Bresadola, Vincenzo Mazzaferro, Andrea Risaliti, Paolo Bertoli, Dario Consonni, Francesco Barretta, Tullia De Feo, Mario Scalamogna

**Affiliations:** 1 U.O. chirurgia Generale e Trapianti di Fegato - Fondazione IRCCS Ca' Granda Ospedale Maggiore Policlinico di Milano, Milano, Italy; 2 UOC Chirurgia Generale e Trapianti d'Organo, Genova, Italy; 3 Chirurgia Generale 2 e dei Trapianti dell'A.O. Ospedale Niguarda Ca' Granda, Milano, Italy; 4 S.S.D. di Chirurgia Epatobiliare e Trapianto Epatico Az. Osp. Di Padova, Padova, Italy; 5 Chirurgia generale, chirurgia vascolare, trapianti d'organo Univ. di Udine, Udine, Italy; 6 U.O. Chirurgia Generale 1 e Trapianto di Fegato, Fondazione IRCCS Istituto Nazionale per lo Studio e Cura dei Tumori di Milano, Milano, Italy; 7 Dipartimento delle Malattie Digestive e Chirurgia Generale, Chirurgia dei Trapianti di Fegato, Rene e Pancreas, Università Politecnica delle Marche, Ospedali Riuniti, Ancona, Italy; 8 Unità Operativa Epidemiologia, Fondazione IRCCS Ca' Granda - Ospedale Maggiore Policlinico and Department of Occupational and Environmental Health, University of Milan, Milano, Italy; 9 Nord Italia Transplant, Fondazione IRCCS Ca' Granda Ospedale Maggiore Policlinico di Milano, Milano, Italy; 10 Department of Clinical Sciences and Community Health, University of Milan & Epidemiology Unit, Department of Preventive Medicine, Foundation IRCCS Cà Granda Ospedale Maggiore Policlinico, Milan, Italy; University of California Los Angeles, United States of America

## Abstract

This study is the largest Italian survey on liver retransplantations (RET). Data report on 167 adult patients who received 2 grafts, 16 who received 3 grafts, and one who received 4 grafts over a 11 yr period.

There was no statistically significant difference in graft survival after the first or the second RET (52, 40, and 29% vs 44, 36, and 18% at 1,5,and 10 yr, respectively: Log-Rank test, p = 0.30).

Survivals at 1, 5, and 10 years of patients who underwent 2 (n = 151) or 3 (n = 15) RETs, were 65, 48,and 39% vs 59, 44, and 30%, respectively (p = 0.59).

Multivariate analysis of survival showed that only the type of graft (whole vs reduced) was associated with a statistically significant difference (HR = 3.77, Wald test p = 0. 05); the donor age appeared to be a relevant factor as well, although the difference was not statistically significant (HR = 1.91, Wald test p = 0.08).

Though late RETs have better results on long term survival relative to early RETs, no statistically significant difference can be found in early results, till three years after RET.

Considering late first RETs (interval>30 days from previous transplantation) with whole grafts the difference in graft survival in RETs due to HCV recurrence (n = 17) was not significantly different from RETs due to other causes (n = 53) (65–58 and 31% vs 66–57 and 28% respectively at 1–5 and 10 years, p = 0.66).

## Introduction

Liver transplantation (LT) can be described as the most revolutionary and challenging surgical technique of the twentieth century. At present, approximately 6000 LT are performed each year both in Europe [1] and in the U.S [2]. LT is now a procedure applied all around the world [3], [4]. However, this surgical option has become a victim of its own success as liver transplantation cannot be performed in all patients who need it. Because of the persistently small number of cadaveric organ donors, and the consequent impossibility to satisfy all needs, the number of patients on the waiting lists continues to rise. Many patients die while waiting for the transplant.

The discrepancy between organ availability and organ necessity is also exacerbated by the circumstance that certain patients need a second liver transplant. Therefore, in a situation of relative organ shortage, our aim was to identify the risk factors related to survival after retransplantation (RET) in a 11–year multicenter Italian study.

Aims of the study were to:

1. calculate graft and patient survival rates after RET
2. compare graft survival rates after the first and the second RET
3. identify the risk factors for the loss of grafts after the first RET.

## Patients and Methods

The study was notified to the Ethics Committee of the Institutions where the data were collected, specifically to the Comitato Etico della Fondazione IRCCS Ca' Granda – Ospedale Maggiore Policlinico. According to the Italian laws (Gazzetta Ufficiale, serie generale, n.76, p. 67–74). No specific request and patient approval are needed for retrospective studies.

Informed consent was obtained as usual for medical, surgical, radiological treatments, not specifically for this retrospective study. Patients gave written consent for every procedure performed in the hospitals including treatment of data for medical purposes.

We collected data on liver RETs performed over 11 a yr period (from 1998 to 2008 included) in transplant centers referring to the Nord Italia Transplant (NIT), the north-Italian organ allocation agency. Eight Transplant Centres were included in the study: Ancona, Genova, Milano Niguarda, Milano Policlinico, Milano Istituto dei Tumori, Padova, Udine S. Maria, and Udine Policlinico. The data collection ended in may 2009. 184 liver RETs in adult patients were studied, including 167 second transplants, 16 third transplants, and 1 fourth liver transplant. The mean follow-up time of the graft after RET was 2.3±2.9 years (median 0.7, range of 0–10.8). The mean follow-up time of patients since the first liver transplant was 4.1±4.5 years (median 2.8, range of 0–22.5).

Continuous and categorical data, referring to the recipient, the donor and the transplant, were collected.

The donor variables included age (years), gender, plasma AST (Aspartate aminotransferase) (UI/L), ALT (Alanine aminotransferase) (UI/L), GGT (gamma -Glutamyltransferase) (UI/L), and sodium (mEq/L); the recipient variables were age (years), gender, serum creatinine (mg/dL), bilirubin (mg/dL), INR (International Normalized Ratio), AST (UI/L), ALT (UI/L), Albumine (g/dL), platelets (n×10^6^/L), MELD (Mayo End-Stage Liver Disease)score, MELD in categories (<15/15–24/25>), UNOS (United Network for Organ Sharing) Status (1, 2a, 2b,3).

The transplant variables included the interval of ischaemia (minutes), the type of transplanted graft (whole graft/partial), extracorporeal circulation (yes/no), the indications for LT, the causes of RET (HAT – Hepatic artery Thrombosis-/PNF –Primary Non Function-/REJ (Rejection)/REC (Recurrence)/Other), the interval (days) from the previous LT (0–8/8–30/30–180/180–365/> 365), the pre-existing graft (whole graft/partial).

The Kaplan-Meier method was used to estimate survival of patients and grafts after RET in relation to the number of transplantations. The Log-Rank test was performed to evaluate the difference between the graft survival after the 1st RET and the graft survival after the 2nd RET; it was also used to compare early and late RETs and the survival after the 1^st^ late RET (performed at least 30 days after LT) with a whole graft, carried out for recurrent HCV (Hepatitis C Virus)cirrhosis vs other indications.

Then we performed an univariate analysis of various parameters in relation to graft survival after first RET, using the Kaplan-Meier estimator and the Log-Rank test for categorical variables and PH Cox model and Wald test for continuous variables. We considered variables of interest those with p-value<0.1.

Finally, the data statistically significant at the univariate analysis, were evaluated in a multivariate analysis with a PH Cox regression model. In this analysis p≤0.05 was considered statistically significant.

## Results

Patients and RETs characteristics are shown in Table 1.

**Table 1:** Donor, transplant and recipient charachteristics in 184 retransplantations as continuous or discrete data.

Continuous Variables	n	mean±SD
**DONOR**		
Age (years)	184	45±16
AST/GOT (IU/L)	154	60±64
ALT/GPT (IU/L)	157	43±48
Gamma GT (IU/L)	123	46±63
Na (mEq/L)	155	147±8
**TRANSPLANT**		
Ischemia time (hours)	184	7.5±2.5
**RECIPIENT**		
Age	184	48±12
Creatinine R (mg/dL)	134	1.7±1.1
Bilirubin R (mg/dL)	137	17.6±15.3
INR R	127	1,7±0,7
MELD Score	132	24±8
AST/GOT R (UI/L)	97	742±1615
ALT/GPT R (UI/L)	120	736±1354
Albumin R (g/dL)	66	3±0,5
Platelets R	118	90.847±76890
**Categorical Variables**	n	%
**DONOR**		
Gender	184	
M	102	56
F	82	44
**TRANSPLANT**		
Graft	184	
Whole	170	97
Partial	14	3
ECC	113	
yes	20	17
no	93	83
**RECIPIENT**		
Gender	184	
M	131	71
F	53	28
Indication 1^st^ LT	167	
Virus rel. cirrhosis	76	45
Alcoholic cirrh.	9	5
Colestatic cirrh.	15	9
HCC	23	14
FHF	8	5
Other	34	20
Missing	2	1
Indication RETX	184	
HAT	42	22
PNF	49	26
Rejection	6	3
Recurrence	10	5
HCV recurrence	19	10
Other	26	14
Missing	32	17
Days from previous LT	184	
0–8	52	28
8–30	37	20
31–180	36	19
181–365	12	6
366+	47	25
MELD Score	132	
<15	22	16
16–24	42	32
25+	68	52
Previous Graft	183	
Whole	144	78
Other	39	21
Status UNOS	184	
1	72	39
2a	80	43
2b	23	12
3	9	5

AST means serum glutamic oxaloacetic transaminase; ALT, alanine transaminase; ECC, Extracorporeal circulation; LT, Liver Transplantation; HCC, Hepatocarcinoma; FHF, Fulminant Hepatic Failure; HAT, Hepatic Artery Thrombosis; PNF, Primary non Function; HCV, hepatitis C Virus; INR, International Normalized Ratio; MELD, Mayo End stage Liver Disease; UNOS, United Network for Organ Sharing.

Overall survivals of patients who underwent RET were 65, 48, and 38% at 1, 5, and 10 years respectively. The 1, 5 and 10 yr survivals of patients who underwent 2 (n = 151) and 3 (n = 15) transplantations were 65, 48 and 39% and 59, 44 and 30% respectively (p = 0.59). The 75% of deaths occurred within 6 months from RET.

The overall graft survival respectively at 1, 5 and 10 years after RET (n = 184) was 51, 40 and 29%, respectively. Graft survival after the first RET (n = 167) at 1, 5, and 10 years was not significantly different (p = 0.30) from survival after the second RET (n = 16): 52, 40, and 29% vs 44, 36, and 18% respectively.

The univariate analysis of factors correlated with 1, 5 and 10 yr survivals is shown in Table 2 for categorical values and Table 3 for continuous values. Several factors reached statistical significance including donor age, serum GOT/AST, time interval < or >30 days, type of graft, ischemia time, recipient age, UNOS state, MELD Score, serum creatinine, platelets count.

**Table 2:** Distribution of the category variables in first retransplantations in relation to survival prospects, by means of univariate analysis (Kaplan-Meier, Log-Rank test).

Categoric variable	Categories			% Survival			Log-Rank test
		N	n	1 y	5 y	10 y	P value
Retransplants 1	Whole series	167		52.1	40	29.3	
Donor's Age (years)	≤60	167	133	57.9	47.1	34	<.001
	61+		34	29.4	13.7		
Previous graft	Whole	167	130	53.8	43.8	30	.14
	Partial		37	45.9	27.9		
Extracorporeal circulation	No	102	85	61.2	48.7	21.5	.29
	Yes		17	41.2	41.2	30.9	
Interval between 1st LT and 1st RETX (days)	≤30	167	78	43.6	28.5	23.7	.02
	30+		89	59.5	49.9	32	
Ischemia time (mnt)	≤720	167	162	65.3	48.1	38.7	.06
	721+		5	40	40		
Gender R	M	167	121	52.8	39.2	33	.87
	F		46	50	41.8	28.2	
Age R	≤55	155	105	56.1	46.1	34.4	.08
	56+		61	45.9	30.5	22.9	
Graft	Whole	167	155	56.1	43.1	31.6	<.001
	Partial		12	0			
Indication for RETX	HAT	138	38	62.5	47.4	47.4	.41
	PNF		44	50	26.1	26.1	
	Rejection		5	60	60		
	Recurrence		10	40	40		
	HCV recurrence		17	64.7	58.2	38.1	
	Other		24	58.3	58.3	29.2	
UNOS	1	167	63	39.6	21.2		.003
	2a		74	55.3	45.4	37	
	2b		21	71.4	66.3	66.3	
	3		9	66.7	55.6	27.8	
MELD	≤15	121	20	85	62.2	62.2	.01
	16–24		38	65.8	54.7	49.2	
	25+		63	49.2	36.3	0	
Creatinine (mg/dL)	≤1.2	124	51	76.5	60.1	48.1	.007
	1.3–2.5		50	56	48.8		
	2.6+		23	39.1	21.7		
PLT	≤50000	107	48	47.9	33.4	33.4	.02
	50001+		59	69.4	54.9	38.9	

**Table 3:** Distribution of the continuous variables in donors and recipients for first retransplantations, with the results from the Cox univariate analysis, in relation to the 1–5–10 years graft survival.

	variable	N	RR	Wald test (p-value)
DONOR	GOT/AST (IU/L)	140	.996	.07
	GPT/ALT (IU/L)	143	.998	.41
	γ –GT (IU/L)	112	1.001	.55
	Sodium (mEq/L)	141	1008	.51
RECIPIENT	T. Bilirubin (mg/dL)	126	1.003	.68
	GOT/AST (IU/L)	87	1	.34
	GPT/ALT (IU/L)	110	1	.77
	INR	118	1.173	.33
	Albumine (g/dL)	58	.741	.32

Result of a multivariate analysis on 10 variables selected after univariate analysis, including 94 observations are shown in Table 4. Only the donor's age (>61years) and the type of graft (partial grafts) showed a statistical significance.

**Table 4:** Results of multivariate cox regression analysis performed on selected variables related to graft failure after first liver retransplantations.

Parameter	Hazard Ratio	95% Hazard Ratio Confidence Limits		P-value
Donor's Age ≤60 years	1.00			
Donor's Age ≥61 years	1.91	0.92	3.94	0.08
Whole Graft	1.00			
Partial Graft	3.77	0.99	14.36	0.05
MELD Score 0–15	1.00			
MELD Score 16–24	2.32	0.65	8.25	0.19
MELD Score ≥25	1.75	0.51	5.99	0.36
UNOS Status 1	1.00			
UNOS Status 2a	0.45	0.14	1.45	0.18
UNOS Status 2b	0.22	0.03	1.24	0.08
UNOS Status 3	0.18	0.02	1.37	0.09
S-creatinine ≤1.2 mg	1.00			
S-creatinine 1,3–2,5 mg	1.37	0.65	2.88	0.39
S-creatinine ≥2.6 mg	1.53	0.59	3.95	0.38
T-ischemia time <12 hrs	1.00			
T-ischemia time ≥12 hrs	1.95	0.20	18.55	0.55
Recipient's Age <56 years	1.00			
Recipient's Age≥56 years	1.15	0.57	2.31	0.67
Platelet count <51000/mm3	1.00			
Platelet count ≥51000/mm3	0.64	0.31	1.29	0.21
Interval from prev. LT≤30 days	1.00			
Interval from prev. LT >30 days	2.441	0.782	7.62	0.12
Donor's AST	0.997	0.991	1.00	0.40

**Figure 1:**
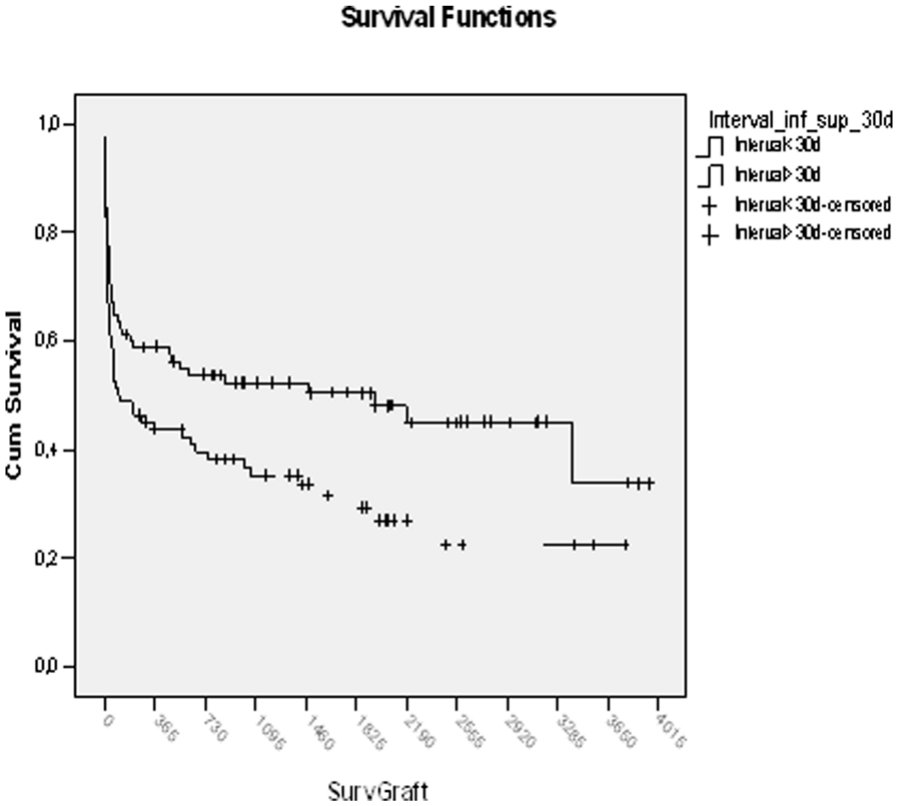
Graft survival after early or late primary Retransplantations.

The analysis of 1-year graft survival, divided on the basis of interval after the previous transplant - (0–8, 9–30, 31–180, 181–365 days and over 1 year), showed a trend to worse outcomes for early RETs relative to late RETs (Fig. 1) but the difference was not statistically significant (p = .28).

The 1, 3 and 5 years graft survival in 82 primary early RET (<30 days from previous LT) relative to 85 late ones (>30 days) was 45.1,35.3 and 29.4% vs 58.8, 52.3 and 50.4%, respectively, with a statistically significant difference (p = .02);

The 60-day graft survival according to the Kaplan Meier method in the two groups based on time interval from previous LT, was 56,1% and 67.1 for grafts with short and prolonged interval respectively, with no statistically significant difference (p = .14).

One year graft survival was 45.1 vs 58.8%, respectively, (p = .08); At two years survival was 41.1 vs 53.7 (p = .10), at three 35.3 vs 52.3(p = .04), at five 29.4 vs 50.4(p = .02), at seven 21.5 vs 45 (p = .01).

Therefore, with regard to post-RET survival, the time interval from previous LT has no statistical relevance during the perioperative time, or within the 1st year after LT but the difference appears to gain significance 3 years after primary RET.

The univariate analysis of usual continuous and categorical variables related to 3 years graft survival identified as important factors banded (0–15, 16–25, over 25) MELD score (p = 0.009), UNOS status (p = 0.01), donor AST (p = .01), recipient serum creatinine (p = .02), time interval from previous LT (p = .02) but not donor age (banded as age<40, 41–70, over 70 years).

The multivariate analysis performed with factors that had a statistical significance on univariate analysis showed only MELD score <15 as an important variable for 3 years survival (p = .03).

Finally, 1–5–10 years graft survival of late RETs (interval>30 days) for HCV recurrence with a whole graft (n = 17) compared to RETs performed for other causes (n = 53) did not show any statistically significant difference (65–58–31% vs 66–57–28% respectively: p = 0.66).

## Discussion

In recent years, LT outcome is progressively improving. As for the December 2010 ELTR (European Liver Transplant Registry) data (downloaded on July 2012), the 1-year survival post LT for adult patients in Europe was 84% from 2000 to 2004 and 85% from 2004 to 2010, while in the U.S was 87% [2].

Despite the progressive improvement in survival, a proportion of liver transplants fail. Consequently, in these cases RET is the “only alternative to death” [5].

In the U.S. the number of RET is approximately 500 per year that is about 8% of transplants [6]. This rate is decreasing.

The causes of RET are highlighted in the 1988–2010 data of the ELTR concerning 6,233 RETs [1]. Whereas within the first weeks after LT, PNF and technical factors have the greater impact, at later intervals, graft rejection and non-cancer recurrences (e.g. hepatitis) are predominant. According to the study of Kashyap [7] published in 2001, concerning transplanted patients from 1986 to 1998 at the University of Pittsburgh, the causes of graft failure that required a second transplant were PNF (32.2%), followed by HAT (27.6%), chronic rejection (14.5%), and primary disease recurrences (5.5%).

A study [5] carried out on 157 RETs performed from 1989 to 2003 showed a sharp decrease in RET due to rejection. In the same study, the rate of RET due to early graft failure (INF - Initial non-function) and HAT were, conversely, increasing.

According to a recent multicenter study by Stange [8] the overall incidence of HAT is less than 5% but the overall mortality rate after a diagnosis of thrombosis is 55% with an 80% rate of RET. This probably depends on two factors: 1) the use of older donors with arteries in a worse condition and 2) on the increased use of reduced livers such as “split right” grafts with hazardous vessels. The increasing rate of RET due to early graft failure (PNF - primary non-function or INF - Initial Not Function -) can also be explained by the larger use of the so-called “marginal donors” [9], [10]. In our study, PNF was the primary indication for RET (32.2%) followed by HAT (27.6%); HCV recurrence was responsible for 12.5% of our RETs.

The overall survival of our patients who underwent RET was 64.6% at 1 year and 47.8% at 5 years. They were mainly patients who underwent two transplants (n = 151) whereas only a small number underwent three transplants (n = 15). In our series, the survival of patients undergoing two transplants was 65, 48 and 39% at 1, 5 and 10 years while for ELTR was 71, 60 and 47%. The Italian data, obtained over the last 10 years, appears worse than those from ELTR data considering RETs from 1988 to 2010.

Consistent with the European data [1], mortality after RET in our series confirms that the most critical period lies within the first 6 months: indeed, 75% of deaths occurred within 6 months from RET.

As reported by ELTR [1], graft survivals after the first RET at 1, 5 and 10 years were 58, 46 and 37%, respectively, whereas our corresponding rates are 52, 40 and 29%. Even in this case, graft survival rates after the first RET in Italy appear lower than those reported in the European study. The cause of this discrepancy might probably emerge only through comparison of data that unfortunately are not available: MELD score, UNOS status, type of grafts, donor and recipient ages. These are all factors that should be considered but that it is not possible to extrapolate from ELTR data.

The European data show a statistically significant difference in graft survival after the first relative to the second RET, with a worse survival after the second transplant. In our series, we observed a similar trend but the difference did not reach statistical significance probably because of the limited number of patients who underwent the second RET.

RET is often a surgically demanding, very expensive procedure [11]. In order to assess the risk associated with a RET, in our univariate and multivariate analyses we studied several parameters including interval after the previous transplant, the UNOS status, the MELD Score and the indication to RET.

The importance of the interval from the previous transplant has been addressed by several authors [12], [13], [14], [15] pointing out that early RETs (range <30 days) have worse outcomes than later ones. Onaca [16], in a small study (44 cases) investigating RETs, carried out at about 2 years after the first transplant, showed a better 2-year survival for “early” (81%) than for late (50%) RETs. In our series this result is ambiguous: indeed, despite there are better long term survivals (Log-Rank test, p = . 02) for late relative to early RETs, this difference becomes evident only after three years from RET. Consequently, we believe that early and late RET are both justified by survival results.

Moreover, because of reported conflicting data concerning RET for HCV recurrence or for other causes, we explored this issue only considering late whole grafts and no difference in survival was found.

The UNOS Status (a classification in four classes of patients according to their clinical conditions before surgery) and the state of emergency related to RET, have been correlated to RET outcome by several authors [11], [14]: the worse results were associated with greater emergency. This observation was confirmed by our study, where the UNOS status was statistically significant at univariate analysis of data on the first RETs (Log-Rank test, p = .003).

Recent observations suggests that the MELD score represents a good prognostic predictor of survival after RET. Ravaioli et al. [17] in 2004 separated their series of RET (n = 87) according to the MELD score at the time of RET. Patients were devided into two groups based on a MELD score >25 and <24. Graft and patient survivals were significantly lower in the first relative to the second group, validating the MELD score as an indicator of survival after RET. Yao e Onaca [16] obtained similar results. In our study, separation of patients in three groups on the basis of MELD scores (<16, 16–24, >25) showed a statistically significant difference in global survivals (Log-Rank test, p = . 02) of patients with different MELD scores. Patients in the higher score groups had the worse survival.

Some authors have also found a correlation between RET indications and subsequent survival. Recent studies [5] point out that PNF and HAT are associated with worse survival. Recurrence of primary disease has intermediate effects. RET for chronic rejection and ischemic biliary lesions have the best survival. In our study, we found no statistically significant association between RET graft survival and indications to RET.

In the attempt to develop prognostic scores, other factors related to prognosis of RET have been studied by several authors. The best known studies are those from Facciuto (48 cases) [19], Meneu (122 cases) [20], Azoulay (139 cases) [17], Rosen (281 cases) [21], Bilbao (74 cases) [22], Yao (40 cases) [18], Linhares (139 cases) [14], and from our group (35 cases) [23]: the most frequently considered factors in the univariate analysis are age, high values of serum creatinine and bilirubin in the recipient, the urgency, the cold ischemia time.

All these variables, in addition to those reported above (UNOS status, MELD score, interval from the previous transplant), were likewise significant in our study: donor age over 60 years, a total ischaemia time longer than 12 hours, recipient age over 56 years, recipient creatinine greater than 2.6 mg/dL, a platelet count less than 50,000, the type of graft used with increased risk for partial grafts, reached statistical significance at univariate analysis.

Although most of the data refer to the recipient, the multivariate analysis emphasizes the importance of two donor factors: 1) type of RET graft (p = .05) - partial grafts in our series did not reach one year of survival and 2) the donor age (p = .08). Although donor age was not statistically significant at multivariate analysis, it clearly deserves consideration.

Our data, therefore, show that survival after the first liver RET mainly depends on the clinical conditions of the recipient but donor factors may likewise have great influence.

In our study we focused our attention on graft survival rather than on patient survival. We believe that, in an era of shortage of organs, though the importance of a single human life, it is an ethical duty to self-limit the use of multiple grafts for a single patient. Despite the good patient survival results reported by some authors [24] after repeated RET, graft survival data must be always considered and a rare resource should not be wasted.

## Conclusions

This is the largest Italian study concerning liver RET.

In our series, graft survival at 1 year after RET is 51%. RET may be considered a waste of a precious resource that could be used for first transplants. However, in our series, the 1-year survival of patients undergoing two transplants is 65%; based on this observation a RET appears to be an acceptable option.

A different approach to early and late RETs does not seem to be justified. Infact though late RETs have better results on long term survival, no statistical difference is found in early results, till three years after RET. So denial of a RET to a patient in the first 30 days post LT is not ethical. As legitimacy of late RETs is established, candidates for RET cannot be selected on the basis of ethiology: graft survivals after late RETs showed no statistically significant difference between patients with HCV recurrence and patients with other indications.

Univariate analysis of factors associated with survival after RET highlights the importance of known factors: they concern both the recipient (UNOS status, MELD score, serum creatinine, platelet number, age), and the donor/transplant (donor age, SGOT, type of graft, ischemia time). At multivariate analysis the recipient's variables lose their significance while the type of graft (whole or reduced) remains significant and the donor age (above or below 60 years) lies close to statistical significance.

According to our study the decision to perform a RET, should be primarily based on the clinical characteristics of the recipient, but the type of graft and the donor age should be considered as factors of substantial importance.
